# The Connecticut Valley and Massachusetts Union Meeting

**Published:** 1888-10

**Authors:** S. G. Stevens

**Affiliations:** Evans House, Boston, Secretary of Committee on Motors


### THE CONNECTICUT VALLEY AND MASSACHUSETTS UNION MEETING.

It was our first visit to the Hub, and we were cordially greeted and entertained throughout our stay. The meeting was a most decided success in a social as well as in a scientific way. The people of New England surely are adepts in the art of entertaining. It is said that they make acquaintances slowly, but never forget a friend. With the remembrance of the pleasant time had by them as the guests of the Montreal Society last year, they planned an elaborate and thoroughly enjoyable return visit. The weather was auspicious the entire week. Every minute of which was occupied, either with clinics, papers, exhibits or excursions, each vieing with the other to claim the time of those in attendance. The work of the several committees was thoroughly done, not a single hitch occurring in the entire programme.

The social part of the programme consisted of first, an informal reception at the Brunswick, Tuesday evening, followed by an elegant lunch. The hosts, with their ladies to the number of 100, received the Canadian dentists and their ladies, numbering 40 or more. The whole evening was spent in a way calculated to give all hands a cordial acquaintance with each other. The visiting dentists present were Drs. S. J. Andres, J. A. Bozine, J. Robert, G. W. Lovejoy, Alfred McDearmid and A. Brosseau of Montreal; A. W. Hyndman and L. W. Dowlin, Sherbrooke, P. Q.; G. H. Weagant, Cornwall, P. Q.; Beacock, Rockville, Ont.; Coggran, Quebec; C. N. Pierce and W. X. Sudduth of Philadelphia; H. B. Noble and D. P. Hickling of Washington, D. C.; J. B. Littig, E. A. Bogue, G. S. Allan and M. L. Rhein of New York city; C. D. Cook of Brooklyn; J. B. Prescott of Manchester, N. H.; J. W. Curtis of Brunswick, Me.; Thomas Fillebrown and the venerable E. Bacon of Porland, Me.; L. C. Taylor and J. McManus of Hartford, Conn.; F. Searle and C. T. Stockwell of Springfield; W. H. Jones of Northampton, Mass.; J. H. Kidder of Lawrence, Mass.; E. S. Gaylord of New Haven, Conn.; and William Barker of Providence, R. I. After dinner speeches were dispensed with and at a seasonable hour, as befitted the members of a dignified and scientific calling, the guests retired to rest feeling on good terms with their hosts.

All members and guests were supplied with badges that proved an open sesamé to many points of interest, including the Museum of Fine Arts, Boston Society of Natural History, and the Cyclorama of Gettysburg and Bunker Hill. These the guests were enabled to see at odd times.

Wednesday afternoon cars were in readiness in front of the Brunswick for an excursion to Cambridge. Under the guidance of Drs. Andrews and Taft, of Cambridge, the party visited the Hemenway Gymnasium, where a short time was spent in examining the numberless appliances for physical training. On departing we were given a peep into the "trophy room," where the pennants and emblems of Harvard's atheletic victories are sacredly preserved. Memorial Hall was next visited. It was erected in the memory of Harvard's graduates who fell in the late rebellion. It is divided into chapel and mess-room, which latter is a place of special interest to Harvard's population, because of the fact that here the more substantial nourishment is dispensed. From here the party were taken to see the library, chemical laboratories, and the collection of minerals in Boylston Hall. A short visit was next made to the

Agissiz Museum, and afterwards to Peabody Museum of American Archæology and Ethnology. The party was warmly welcomed by Prof. F. W. Putnam in a few, well-chosen words. All were highly pleased and captivated by the genial professor's untiring efforts to explain the object and contents of the museum, and his kind attention will long be remembered as an important part of the pleasant excursion. The collection of skulls is very large, and the teeth of various ages were examined with much interest by the visitors. There is, perhaps, no finer collection in existence, and all felt that many days could be profitably spent in their examination. The hour of departure came only too soon, and we tore ourselves away from viewing decay in *facto* to study it in *causo* in the evening, by listening to Dr. Allan's paper and looking at the beautiful slides exhibited by means of the stereoptican, and which formed one of the very many pleasant features of the week's entertainment.

DOWNER'S LANDING AND THE CLAM BAKE.

Thursday afternoon a trip down the harbor had been planned. Again cars were in readiness in front of the Brunswick, which were soon filled by some three hundred of the members, guests, and their ladies. After a circuitous drive past the most prominent buildings the Battery wharf was reached, and all boarded the steamer "Stanford," and we glided out into the pride of all Boston. In the inner bay the white caps were still running, the result of a storm the previous night; and before Fort Warren was reached more than one face had grown a shade or two paler. A landing was made and a visit to the fortifications was greatly appreciated. The stiff breeze on *terra firma* served to steady the nerves of those who had found it rather uncomfortable on the water. The next stop was at Downer's Landing, where a clam bake had been provided. Downer's Landing is the property of the Downer estate, Mrs. Dr. Littig, of New York, is one of the heirs. The genial doctor and family spend their summers there, and were present to welcome the visitors. It is quite a favorite temperance resort, no liquors of any sort being allowed on the premises. The buildings and grounds are well kept, and everything done to make the visitor comfortable while there. The dinner over, some time was spent in visiting the grounds and quietly chatting. The return whistle came all too soon. It had been intended to take in some of the principal harbor public institutions, but on account of the rough sea it was given up. The chairman of the committee on entertainments, Dr. Shepherd, said it was a pleasure excursion, and if a single person would be discomforted by exposure it would be given up. He evidently found the single individual, for we came directly in landing about 7 P.M., feeling that an enjoyable afternoon had been spent.

Perhaps one of the most pleasant features of the union meeting was the presentation to Dr. Flavius Searle of a beautifully bound copy of the full programme of the evening session of the Connecticut Valley Dental Society, held at Springfield, Mass., October 27, 1887. It will be remembered that a surprise had been prepared for Dr. Searle, and was carried out with entire success, as follows:

After the reading of his paper, entitled "Retrospect, or Some Reflections Based upon Experiences and Observations of Fifty Years of Dental Practice," the discussion took the form of a series of congratulatory speeches and the reading of letters from those who were unable to attend. These letters, together with the speeches made, some forty in all, were preserved by the secretary, Dr. Maxfield, who, in accordance with a resolution passed by the Society, had had them handsomely bound in a beautifully typewritten volume, bearing upon its cover the inscription: "Presented to Dr. F. Searle by the Connecticut Valley Dental Society." Dr. Searle is justly proud of the gift, as he well may be. It seldom comes to members of the profession to reach such a ripe experience in active practice, and the words of esteem so freely extended, prepared in such permanent and artistic form, cannot fail to become a valuable heirloom in the Searle family—a good name is more to be preferred than riches, says the good book—and Dr. Searle surely has one, if the testimony of his fellow practitioners can be relied upon.

THE EXHIBIT.

Through the untiring efforts of Drs. Horatio C. Merriam and W. E. Page, Secretary of the Committee on Exhibits, a most instructive exhibit was brought together. Dentists, by reason of the sedentary nature of their occupation, seldom get out among the manufacturers; but depend upon having their supplies brought to their doors by the dealers. As a consequence, a class of middle men who, in most instances are manufacturers also, has been developed. They have put drummers upon the road, and sample cases are carried into every hamlet and village where there is any probability of selling a bill of goods. This arrangement has been agreeable and advantageous to the profession, as well as profitable to the dealer. A dentist's time forms his most valuable stock in trade. His working hours are limited, and his earnings go on only when *he* is attending to business; consequently he has very little time to run around looking up bargains, and as a result the profession has come to depend upon a few dealers for their supplies. And why a few? One would naturally suppose that supply and demand would, to a great extent at least, govern competition in the open market, and so it would in the dealings between any other profession and the trade. But in the matter of dental supplies an organization, offensive and defensive, has been formed by the leading dealers and manufacturers to keep up prices and cripple competition.

A prominent feature in American character is the desire to drive a sharp bargain. To be told that he cannot have an article any cheaper because the price has been fixed by "The combination," naturally irritates him; and while he may for the time being acquiesce, he naturally vows to get even some time or other. When a man's inherent right to purchase his goods in the open market is taken away from him he naturally rebels. Such has been the history of the operation of the combination in its effect upon the profession, and which resulted to produce the display shown at the Boston meeting. The profession gave expression freely to its feeling in the matter, and it remains to be seen what will be the outcome. It was a high pressure meeting all the way through. It took a great amount of energy to get up the exhibit, which was nothing more or less than a remonstrance against the combination, and was so considered by every dentist present at the meeting. As an independent journal it becomes our duty to record all matters of interest to the profession, and this surely was a marked feature of the Boston meeting, and one that forced itself upon the attention of all who were present. Whether any material good will result therefrom we are unable to say. At any rate for the time being the producer and consumer were brought more directly together than ever before. Much depends as to whether those who were present will follow up this advantage by keeping their goods before the profession by advertising them in the journals that are open to receive their advertisements.

THE CLINICS.

The clinics also formed a prominent feature of the meeting, and were fully appreciated. The forenoon of Wednesday was occupied by the operations of the following named gentlemen:

Dr. W. P. Cooke, Boston, Filling with No. 30 Gold Foil.

Dr. D. M. Clapp, Boston, Combination Fillings.

Dr. D. D. Peabody, Stoneham, Mass., Clinic with Back Action Mallet.

Prof. J. Bond Littig, New York City, Porcelain Tips.

Dr. G. F. Eames, Boston, Regulating by Replantation.

THURSDAY.

Dr. E. S. Gaylord, New Haven, Conn., Plastic Gold Filling, Ivory Points.

Dr. Geo. C. Ainsworth, Boston, Soft Gold Filling.

Dr. J. E. Waitt, Boston, Progressive Finishing.

Dr. C. Frank Bliven, Worcester, Mass., Filling with Electric Mallet.

Dr. S. S. Stowell, Pittsfield, Mass., Copper Amalgam Filling.

FRIDAY.

Dr. E. C. Leach, Boston, Porcelain Tips.

Dr. D. F. Whitten, So. Boston, "Preparation of Approximal Surfaces."

Dr. J. G. W. Werner, Boston, "Marginal Cavities."

The following Motors suitable for dental purposes were on exhibition and most of them were in operation:

ELECTRIC.

*The Baxter Electric Manufacturing & Motor Co.*, of Baltimore, Md., through their agent, Mr. Frank Ridlon, of 178 Devonshire Street, Boston, exhibited their small Electric Motor.

*The Geo. F. Card Manufacturing Co.*, of Cincinnati, Ohio, showed small Electric Motors.

*The C. & C. Electric Motor Co.*, 90 South Fifth Avenue, New York, exhibited their Electric Motors.

*The U. S. Electric Light & Battery Co.*, 161 Milk Street, Boston, had on exhibition their Batteries with Motors for dental and other purposes requiring two-horse power and less.

*The S. S. White Dental Manufacturing Co.* presented the Detroit Electric Dental Motor.

*The Kellar Medicine Co.*, of Fort Wayne, Ind., through their agent, Mr. J. R. Baseman, showed the Woolley Magnetic Dental Engine, which was pronounced perfection by many.

GAS.

*The Economic Gas Engine Co.*, 34 Dey Street, New York, brought out a one-eighth horse-power Engine.

WATER.

*The Benham Hydraulic Motor Co.*, of Providence, R. I., were on hand with their small Hydraulic Motor, suitable for dental uses.

*The Tuerk Hydraulic Power Co.*, office 12 Cortlandt Street, New York, had on exhibition one of their No. 9 Water Motors.

*The Tuerk Water Meter Co.*, of Syracuse, New York, through B. L. Knapp & Co., 161 Tremont Street, Boston, showed one of their small Dental Motors.

S. G. STEVENS,
Evans House, Boston,
*Secretary of Committee on Motors.*

